# Beyond Fat: Reframing MASLD Through Genetics, Clonal Biology, and Precision Hepatology

**DOI:** 10.3390/ph19081145

**Published:** 2026-07-24

**Authors:** Javier Crespo, Marta Alonso-Peña, Carolina Jiménez-González, Lorena Cayón-Gonzalez, Paula Iruzubieta

**Affiliations:** 1 Cantabria Cohort, Valdecilla Research Institute (IDIVAL), 39011 Santander, Spain; 2 Departmento de Medicina, Universidad de Cantabria, 39011 Santander, Spain; 3 MedicineAI, 28027 Madrid, Spain; 4 Departamento de Anatomía y Biología Celular, Universidad de Cantabria, 39011 Santander, Spain; marta.alonso@unican.es; 5 Clinical and Translational Research in Digestive Diseases, Valdecilla Research Institute (IDIVAL), 39011 Santander, Spainlorena.cayon@idival.org (L.C.-G.); p.iruzubieta@gmail.com (P.I.); 6 Gastroenterology and Hepatology Department, Marqués de Valdecilla University Hospital, 39008 Santander, Spain

**Keywords:** MASLD, MASH, genetics, polygenic risk scores, clonal evolution, pharmacogenomics, precision medicine, PNPLA3, HSD17B13, hepatocellular carcinoma

## Abstract

Metabolic dysfunction-associated steatotic liver disease (MASLD) has traditionally been conceptualized as a predominantly metabolic disorder driven by obesity and insulin resistance. However, recent advances in human genetics have revealed a more complex picture that encompasses germline susceptibility variants, protective loss-of-function alleles, polygenic risk models, and somatic clonal evolution. Since the discovery of PNPLA3 (patatin-like phospholipase domain-containing 3) I148M, multiple loci—including TM6SF2, MBOAT7, GCKR, HSD17B13, MTARC1, GPAM, and CIDEB—have substantially expanded the mechanistic understanding of disease heterogeneity and hepatocellular vulnerability. Recent studies integrating partitioned polygenic risk scores and unsupervised phenotypic clustering suggest that MASLD may be organized into at least two predominant subtypes: a liver-specific subtype characterized by intrinsic hepatocellular susceptibility, and a cardiometabolic subtype associated with systemic metabolic dysfunction and increased cardiovascular risk. Analyses of cirrhotic liver tissue have, in turn, demonstrated somatic clonal expansion of hepatocytes harboring adaptive metabolic mutations, adding an evolutionary dimension to advanced disease. On this basis, we propose an integrated LS/CM/C framework encompassing liver-specific (LS), cardiometabolic (CM), and clonal (C) components. This model offers a conceptual structure that links germline genetics, metabolic heterogeneity, somatic adaptation, and emerging pharmacogenomic strategies. The recent development of genotype-directed therapies targeting PNPLA3 and HSD17B13, together with the approval of resmetirom and semaglutide, further supports the transition toward biologically stratified hepatology. Although prospective validation remains necessary, the convergence of genetics, clonal biology, and targeted therapeutics suggests that MASLD is moving toward an era of precision medicine.

## 1. Introduction

MASLD affects approximately 30% of the global adult population, with an estimated prevalence approaching 25% in Western Europe and steadily increasing rates across Latin America, the Middle East, and East Asia [1,2]. Although grouped under a single clinical designation, its natural history is remarkably heterogeneous: while some individuals remain metabolically stable for decades, others progress toward metabolic dysfunction-associated steatohepatitis (MASH), advanced fibrosis, cirrhosis, or hepatocellular carcinoma, a variability that reflects the complex interaction between metabolic, environmental, and genetic determinants.

In 2008, the PNPLA3 I148M variant was identified as the principal genetic determinant of MASLD [3]. This discovery profoundly reshaped the biological understanding of what was then termed non-alcoholic fatty liver disease (NAFLD) by demonstrating that susceptibility to hepatic fat accumulation did not depend exclusively on obesity, insulin resistance, or type 2 diabetes, but also on genetic variants exerting direct effects on the hepatocyte itself. Over the following decade, the field evolved from the identification of isolated variants toward a substantially more complex genetic architecture composed of germline risk loci, protective loss-of-function variants, polygenic risk scores (PRS), and somatic alterations acquired during advanced disease progression [4,5,6,7].

This conceptual expansion has coincided with an equally remarkable therapeutic acceleration. The approval of resmetirom following the MAESTRO-NASH trial marked the beginning of the first generation of approved therapies for MASH with significant fibrosis [8,9]. Shortly thereafter, the ESSENCE study confirmed the histological efficacy of semaglutide 2.4 mg in MASH [8], building on an earlier phase 2 trial [10]. Around the same time, several therapies targeting PNPLA3 and HSD17B13 entered early clinical development using antisense oligonucleotides (ASO) and small interfering RNA (siRNA), achieving reductions in hepatic messenger RNA approaching 90% [11,12,13]. For the first time, clinical trials in hepatology began selecting patients according to genotype.

At the same time, genetic studies began to challenge the notion of MASLD as a biologically homogeneous entity. In 2024, two independent studies published simultaneously in Nature Medicine identified distinct disease patterns [14,15]. One employed partitioned polygenic risk scores, whereas the other applied unsupervised clinical clustering; nevertheless, both converged on the existence of two predominant patterns: a hepatic one and a cardiometabolic one. This agreement between genetic and phenotypic approaches suggested that the clinical heterogeneity of MASLD may reflect partially distinct biological mechanisms, with potentially relevant implications for therapeutic response.

The cardiometabolic dimension of this model also aligns with the broader cardio-kidney-metabolic (CKM) continuum recently proposed by the American Heart Association, in which the liver, visceral adiposity, vascular inflammation, renal dysfunction, and insulin resistance are integrated within a unified pathophysiological network [16,17]. In this framework, the liver is no longer interpreted merely as a passive target organ of metabolic syndrome, but rather as a central node within systemic cardiometabolic disease.

Despite these advances, genetic stratification has not yet been incorporated into routine clinical practice. The international consensus that replaced NAFLD with MASLD did not include genetic criteria [18], and neither the AASLD nor the EASL-EASD-EASO guidelines currently recommend routine genotyping [18,19]. Diagnostic and therapeutic decisions therefore continue to rely predominantly on fibrosis stage, histological activity, and metabolic comorbidity, while genetics remains largely confined to translational research settings.

This gap between biological knowledge and clinical implementation likely represents the major unresolved tension within the field. MASLD genetics no longer merely identifies susceptibility; it increasingly delineates distinct biological mechanisms, potential evolutionary trajectories, and therapeutically actionable targets. In other areas of medicine—particularly oncology and preventive cardiology—the transition from susceptibility biomarkers toward predictive and pharmacogenomic biomarkers has already transformed clinical trial design and therapeutic selection [20,21,22,23]. MASLD may now be approaching a similar transition.

**Figure 1:**
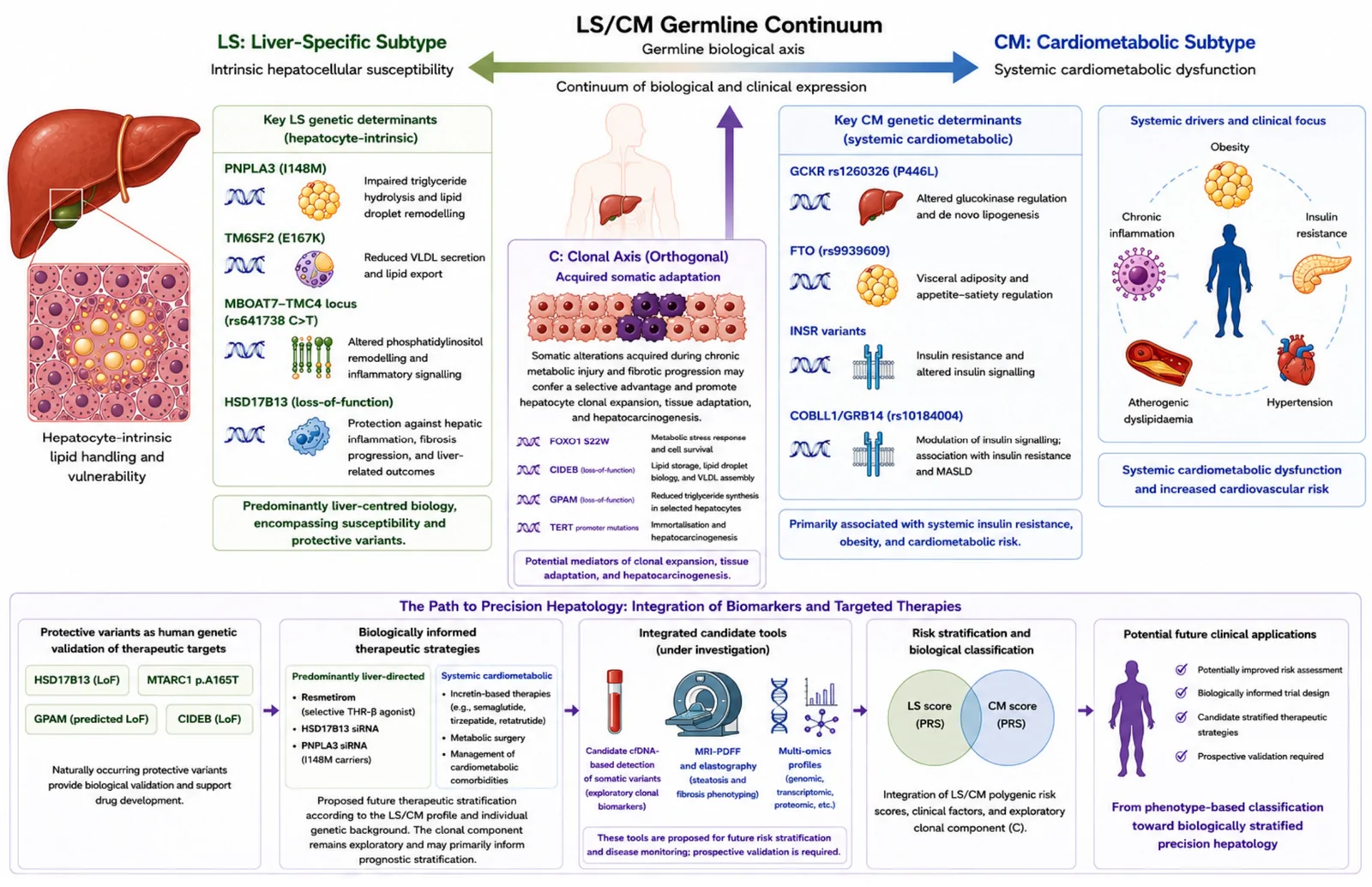
Proposed LS/CM/C biological framework of metabolic dysfunction-associated steatotic liver disease. The horizontal axis represents a germline continuum extending from predominantly liver-specific (LS) biology, characterised by hepatocyte-intrinsic susceptibility and protective variants, to predominantly cardiometabolic (CM) biology, driven by systemic insulin resistance, visceral adiposity, dyslipidaemia, inflammation, and related cardiometabolic dysfunction. The orthogonal clonal component (C) represents acquired somatic adaptation arising during chronic liver injury and fibrotic progression, with selected hepatocyte clones potentially contributing to tissue adaptation and hepatocarcinogenesis. The lower translational layer integrates human genetic validation, biologically informed therapeutic strategies, partitioned polygenic risk scores, imaging-based phenotyping, multi-omic profiling, and candidate detection of somatic variants in circulating cell-free DNA. The LS/CM/C model is intended as an integrative and testable framework for future risk stratification, clinical-trial design, and precision hepatology; its clinical utility requires prospective validation. Abbreviations: cfDNA, circulating cell-free DNA; CM, cardiometabolic; C, clonal; LS, liver-specific; LoF, loss-of-function; MASLD, metabolic dysfunction-associated steatotic liver disease; MRI-PDFF, magnetic resonance imaging proton density fat fraction; PRS, polygenic risk score; siRNA, small interfering RNA; VLDL, very-low-density lipoprotein.

In this review, we propose that MASLD genetics is evolving from a risk-centered paradigm toward a progressively more biologically stratified and pharmacogenomic hepatology. To explore this hypothesis, we review the current genetic architecture through the integration of three interconnected levels: germline risk variants, protective loss-of-function variants, and acquired clonal somatic alterations. On this basis, we propose an interpretative framework composed of two major biological patterns—liver-specific (LS) and cardiometabolic (CM)—together with a potential acquired clonal component (C) associated with advanced disease progression. Finally, we examine how this model could influence the development of genotype-directed therapies, dynamic biomarkers, and future stratified clinical trials, while deliberately maintaining a critical perspective and acknowledging that many of these implications remain hypotheses awaiting prospective validation (Figure 1 and Box 1).

Box 1 **Take-home messages.**

– MASLD may represent biologically distinct disease trajectories rather than a single metabolic disorder.
– Germline and somatic genetics are beginning to redefine hepatology as a biologically stratified medicine.
– A liver-specific (LS) and a cardiometabolic (CM) germline axis capture much of this heterogeneity, but not all of it.
– An orthogonal acquired clonal axis (C) is proposed as an operational hypothesis to accommodate somatic selection in advanced disease.
– The convergence of LS, CM, and clonal biology may shape future therapeutic selection more than histology alone.
– The decisive near-term test is not a new trial but the stratified re-analysis of existing pivotal datasets.

## 2. Genetic Architecture and Biological Complexity of MASLD (Table 1)

The first major genetic signal in MASLD emerged with the identification of PNPLA3 rs738409 (I148M) through genome-wide association studies (GWAS) [3]. This variant displayed an exceptionally strong effect size for a complex disease, with an odds ratio approaching 3 for fatty liver disease and striking ancestral differences in frequency. Subsequent functional studies demonstrated that the mutated protein accumulates on hepatocellular lipid droplets and disrupts lipolysis mediated by the ATGL-CGI-58 complex [24,25]. Mechanistically, I148M is not a simple loss-of-function variant: it reduces intrinsic hydrolase activity, resists ubiquitin-mediated degradation and accumulates on lipid droplets, sequesters ABHD5/CGI-58 and thereby impairs ATGL-mediated lipolysis, and additionally perturbs hepatocellular lipid trafficking and export—features with a neomorphic, gain-of-function character consistent with Table 1.

A few years later, TM6SF2 E167K was identified through whole-exome sequencing studies [26]. This variant was associated with increased hepatic fat accumulation and fibrosis, but paradoxically also with lower circulating concentrations of LDL cholesterol and triglycerides. Mechanistic studies subsequently demonstrated that TM6SF2 participates in VLDL assembly and secretion, whereas the E167K variant promotes intrahepatic lipid retention [27,28]. More specifically, E167K causes protein misfolding and accelerated degradation with a reduced secretion of large, triglyceride-rich VLDL1 particles, producing lower plasma triglycerides together with hepatic lipid retention.

Additional relevant loci were subsequently incorporated into the MASLD genetic landscape, including MBOAT7-TMC4 rs641738 and GCKR rs1260326 (P446L). MBOAT7 was linked to alterations in hepatic phosphatidylinositol remodeling [29,30,31], whereas GCKR modulates hepatic glycolytic and lipogenic fluxes through regulation of glucokinase activity [32].

The subsequent expansion of multi-ancestry GWAS consolidated the polygenic nature of MASLD. Within the Million Veteran Program, seventy-seven loci associated with unexplained chronic ALT elevation as a proxy for MASLD were identified [33]. Later studies integrating MRI-PDFF with electronic health records confirmed additional loci, including APOE, MTARC1, PNPLA2, TRIB1, GPAM, COBLL1, and FTO [34]. Current estimates suggest that the number of robustly associated loci now substantially exceeds forty [7,35].

This genetic expansion also showed that not all variants converge upon identical biological mechanisms. Some predominantly affect intrinsic hepatocellular lipid metabolism—such as PNPLA3, TM6SF2, and MBOAT7—whereas others are more closely linked to visceral adiposity, glucose metabolism, or insulin resistance. These differences progressively challenged the view of MASLD as a single, homogeneous metabolic disorder.

**Table 1:** **Genetic architecture of MASLD within the LS/CM/C framework.** Integrated model of MASLD genetic architecture organised by position in the LS/CM/C framework: germline risk variants (LS and CM axes), germline protective variants, and convergent somatic/clonal alterations. ↑: increase; ↓ decrease.

**A. LS Germline Variants**											
**Variant/SNP**		**Gene/Function**		**Allele Frequency**		**Effect**		**Molecular Mechanism**			**References**
rs738409 (I148M)		PNPLA3/lipid droplet lipase		0.49 HISP; 0.23 EUR; 0.17 AFR		OR 3.12 MASLD; HR ~2 cirrhosis; ↑ HCC		Anomalous gain of function; accumulation on lipid droplets; blockade of ATGL-CGI-58 lipolysis			[3,24,25]
rs58542926 (E167K)		TM6SF2/VLDL assembly		0.07 EUR; 0.03–0.11 global		↑ hepatic fat; paradoxical ↓ LDL		Misfolding and accelerated degradation; ↓ VLDL lipidation; reduced secretion of large, triglyceride-rich VLDL1			[26,27,28]
rs641738 C>T		MBOAT7-TMC4/PI remodelling		0.43 EUR; greater effect in Hispanics		OR 1.2–1.4 NAFLD; ↑ fibrosis		Altered hepatic phosphatidylinositol composition; TLR/inflammation signalling			[29,31,36]
rs2792751/rs10433937		GPAM/mitochondrial glycerol-3-P acyltransferase		Variable		↑ hepatic fat content		Increased triglyceride synthesis; altered mitochondrial function			[34,37]
rs2954038		TRIB1/lipid metabolism regulator		0.32 EUR		↑ MASLD and plasma lipids		Modulation of hepatic expression of lipogenic genes			[7,34]
rs429358/rs7412 (APOE)		APOE/lipoproteins		Variable (E2/E3/E4)		Modulatory effect on MASLD and CVD		Influence on lipoprotein clearance and hepatic deposition			[7,34]
rs2862954		ERLIN1/ER cholesterol homeostasis		Variable		↑ hepatic fat content		SREBP regulation and lipid droplet biogenesis			[34]
rs2228603		NCAN (TM6SF2 locus)		Variable		↑ MASLD by imaging		Co-segregation with TM6SF2 E167K			[34,38]
rs61756425 (PSD3)		PSD3/pleckstrin and Sec7 domain		Variable		Association with MASLD progression		Replicated finding in recent cohorts			[7]
**B. CM germline variants**											
**Variant/SNP**		**Gene/function**		**Allele frequency**		**Effect**		**Molecular mechanism**			**References**
rs1260326 (P446L)		GCKR/glucokinase regulation		0.40 EUR		↑ triglycerides; ↑ hepatic DNL; ↑ MASLD		Increased de novo lipogenesis under hyperinsulinaemia			[32,34]
rs9939609 (FTO)		FTO/adiposity regulation		0.42 EUR		↑ visceral adiposity; ↑ secondary MASLD		Modulation of appetite-satiety; mTOR signalling			[7,33,34]
rs17782313 (MC4R)		MC4R/melanocortin receptor		Variable		↑ adiposity; ↑ MASLD		Hypothalamic appetite regulation			[33,34]
rs10184004 (COBLL1/GRB14)		COBLL1/insulin sensitivity		0.40 EUR		Insulin resistance; MASLD		Modulation of insulin signalling			[14,34]
INSR variants		INSR/insulin receptor		Variable		Insulin resistance		Modulation of insulin cascade			[14,34]
rs5742915 (PNPLA2/ATGL)		PNPLA2/triglyceride lipase		Variable		Adiposity-associated MASLD		Triglyceride hydrolysis			[34]
rs6831256 (ADH1B)		ADH1B/alcohol dehydrogenase		Variable		Modulation of MASLD linked to alcohol intake		Alcohol metabolism; relevant for MetALD			[34]
rs4253772 (PPARA)		PPARA/lipid-metabolism receptor		Variable		Systemic lipid modulation		Regulation of fatty acid oxidation			[34]
rs17231506 (CETP)		CETP/cholesterol transfer		Variable		Systemic lipid profile		CE-TG exchange between lipoproteins			[34]
**C. Protective variants**											
**Variant**	**Gene**		**Frequency**		**Risk reduction**		**Pharmacological target**		**Clinical programme**	**Reference**	
rs72613567:TA (splicing)	HSD17B13		0.26 EUR		30% ↓ liver disease; 17% ↓ HCC		siRNA/ASO anti-HSD17B13		Rapirosiran phase 2; GSK4532990 phase 2b	[12,13,39,40]	
rs2642438 (p.A165T)	MTARC1		0.29 MAF		↓ hepatic fat; ↓ ALT; ↓ liver-related mortality		MTARC1 inhibitor		Preclinical	[37,41]	
LoF heterozygous rs150562387	CIDEB		~1/700		53% ↓ NAFLD; 54% ↓ non-alcoholic cirrhosis		CIDEB inhibitor		Preclinical	[42,43]	
Predicted LoF	GPAM		Variable		↓ MASLD and cirrhosis		GPAM inhibitor		Preclinical	[37]	
Rare LoF variants	APOB		<0.001		Familial hypobetalipoproteinaemia; complex MASLD relation		Indirect target		Preclinical/rare	[7]	
Rare LoF variants	MTTP		Rare		Abetalipoproteinaemia; MASLD modulation		Indirect target		—	[7,34]	
Rare variants	ABCB4/MDR3		Rare		Modulation of cholestatic-MASLD phenotype		Indirect target		—	[7]	
Klotho variants	KL		Variable		Protection against severe MASLD progression		Exploratory target		Preclinical	[44]	
**D. Somatic/clonal alterations**											
**Mutation/gene**		**Finding in advanced MASLD**		**Cohort/methodology**		**Functional significance**		**Clinical implication**			**References**
FOXO1 hotspot S22W		7/29 patients with advanced liver disease (~24%); ≤9 clones per patient		Ng 2021: 1590 whole-genome samples; 34 hepatic samples, microdissection and deep sequencing		Altered insulin-mediated nuclear export; constitutive transcriptional activation; selective proliferative advantage under metabolic stress		Plausible therapeutic target (programme yet to be created); candidate biomarker via cfDNA			[38]
CIDEB somatic LoF		Frequent in MASLD cirrhosis		Ng 2021 somatic whole-genome analysis; consistent with Zeng 2026		Functional equivalent to germline LoF: individual hepatocyte protection plus selective clonal advantage under chronic damage		Integrative LS/C target: only target with convergent germline + somatic validation			[38,45]
GPAM somatic LoF		Frequent in MASLD cirrhosis		Ng 2021 somatic whole-genome analysis		Functional equivalent to germline LoF; reduced triglyceride synthesis in selected hepatocytes		Plausible therapeutic target; partial germline-somatic convergence			[38]
PKD1, PPARGC1B, KMT2D, ARID1A		Millimetre-scale clonal expansions; multiple patterns		Brunner 2019: 482 microdissections; 82 patients with chronic liver disease of various aetiologies		Diverse; chronic liver damage not specific to MASLD		Biomarkers of chronicity/hepatic progression			[46]
TERT promoter		Finding in advanced cirrhosis		MASLD cirrhosis and nodulation analysis		Preneoplastic clonal immortalisation		Marker of HCC transition in longitudinal cohorts			[45,46]

### 2.1. Protective Variants and Human Biological Validation (Table 1)

The identification of protective loss-of-function variants introduced a major conceptual transition within the field. Unlike cellular or animal models, naturally occurring carriers of protective variants provide a unique opportunity to observe the long-term physiological consequences of partial or complete inhibition of a molecular target in humans.

The most paradigmatic example is HSD17B13. In 2018, a truncating variant was described as being associated with protection against chronic liver disease, inflammation, and fibrosis, even in the presence of obesity or classical metabolic risk factors [39]. Subsequent studies demonstrated that HSD17B13 encodes a hepatocellular lipid droplet-associated protein involved in retinoid metabolism [47,48]. This discovery rapidly transformed HSD17B13 into one of the most prominent pharmacogenomic targets in MASLD.

Similar observations subsequently emerged for MTARC1 (mitochondrial amidoxime reducing component 1). Variants such as p.A165T were associated with lower hepatic fat accumulation, reduced ALT levels, and decreased liver-related mortality [37]. Although the precise mechanisms remain only partially understood, MTARC1 appears to participate in mitochondrial metabolism and hepatocellular redox homeostasis [49].

GPAM (mitochondrial glycerol-3-phosphate acyltransferase 1), the rate-limiting enzyme initiating hepatic triglyceride and glycerolipid synthesis, represents a particularly relevant example because it connects lipid metabolism with advanced disease progression. Protective loss-of-function variants have been associated with lower risk of MASLD and fibrosis [37].

CIDEB (cell death-inducing DFFA-like effector B), a hepatocellular lipid droplet-associated protein involved in intracellular lipid storage and VLDL assembly, probably constitutes one of the most biologically distinctive examples in the field today. Heterozygous germline loss-of-function variants reduce MASLD risk by approximately 53% [42].

The conceptual importance of these variants extends well beyond descriptive genetics. In cardiology, naturally occurring protective variants anticipated the therapeutic success of pharmacological inhibitors such as PCSK9 inhibitors [50,51]. The development of therapies targeting HSD17B13 and other hepatocellular pathways now follows a similar rationale: using human genetics as biological validation prior to pharmacological development.

### 2.2. Polygenic Risk Scores and Functional Organization of Risk (Table 2)

As the number of MASLD-associated loci expanded, it became increasingly evident that no individual variant could independently explain the enormous clinical heterogeneity of the disease. This realization drove the development of polygenic risk scores (PRS), constructed through weighted combinations of multiple germline variants with the initial aim of estimating cumulative susceptibility to steatosis, fibrosis, or hepatocellular carcinoma [52,53].

The earliest PRS models primarily integrated PNPLA3, TM6SF2, MBOAT7, and GCKR, demonstrating moderate ability to predict advanced fibrosis and HCC. Subsequent incorporation of additional loci identified through multi-ancestry GWAS progressively improved the biological resolution of these models [33,34].

The most important conceptual advance emerged from the observation that distinct genetic combinations appeared to cluster around partially differentiated biological mechanisms. Certain profiles were dominated by alterations in hepatocellular lipid metabolism, whereas others displayed stronger associations with visceral obesity, insulin resistance, and cardiovascular risk. These findings suggested that the clinical heterogeneity of MASLD may reflect fundamentally distinct biological architectures rather than merely different degrees of severity within a single metabolic disorder.

The cardiometabolic pattern identified by these models also aligns closely with the cardio-kidney-metabolic (CKM) framework proposed by the American Heart Association [16,17], which positions the liver within an integrated pathophysiological network involving visceral adiposity, vascular inflammation, insulin resistance, and renal dysfunction.

Nevertheless, important limitations persist. No PRS in MASLD has yet been prospectively validated as a predictive biomarker of therapeutic response in a pivotal clinical trial. In addition, PRS developed predominantly in European populations demonstrate substantial loss of performance across other ancestries [40,54]. It also remains unclear whether these profiles represent stable biological trajectories or dynamic states modifiable by aging, obesity, weight loss, or pharmacological intervention.

**Table 2:** **Polygenic risk scores and biological stratification in MASLD.** Construction, validation, performance, and operational position of polygenic risk scores with respect to the LS/CM/C framework. The Jamialahmadi [12] partitioned PRS is the first to partition variants by underlying biological pathway, aligning directly with the LS-CM axis.

PRS	Construction	Validation	Performance	Position in LS/CM/C Framework	Reference
PRS-HFC	PNPLA3 + TM6SF2 + GCKR + MBOAT7 + (HSD17B13)	NAFLD cohort 2566; UK Biobank 364,048	OR HCC ~3; OR non-viral HCC ~4	Classical LS core; precursor of partitioned PRS	[53]
PRS	GWAS 66,814 imaging + 624,665 individuals by diagnostic code	Independent cohorts	99th percentile: 2.5–6× risk of MASLD/cirrhosis/HCC	Discriminates risk in both subtypes	[34,35]
Multi-ethnic PRS Thrift	Multi-ancestral	Patients with cirrhosis	HR 2.30 per unit; C-statistic 0.70	HCC predictor in cirrhosis	[55]
Partitioned PRS	Partitioned by hepatic lipoprotein retention	36,394 individuals + 3903 replication	LS/CM bipartition with distinct biological profiles	First PRS operating directly on the LS/CM framework	[14]

### 2.3. Somatic Evolution and Hepatocellular Clonal Adaptation

Advanced liver disease introduces an additional biological dimension extending beyond inherited susceptibility: the emergence of acquired somatic mutations in hepatocytes exposed for years to inflammation, metabolic stress, cell death, and compensatory regeneration.

Brunner et al. demonstrated that the cirrhotic human liver contains clonal hepatocyte expansions harboring acquired somatic mutations shared across multiple clones within the same individual [46]. This work reframed cirrhosis not merely as a fibrotic process, but also as a dynamic ecosystem subjected to continuous selective pressure.

Subsequent studies identified recurrent somatic mutations in metabolic genes within livers affected by advanced chronic liver disease [38]. Among the most relevant alterations were FOXO1 S22W and loss-of-function mutations involving GPAM and CIDEB. These mutations appear to emerge in hepatocytes acquiring adaptive advantages under sustained metabolic stress, potentially promoting cellular survival, reduction in lipotoxicity, or bioenergetic adaptation.

The overlap between protective germline variants and acquired somatic mutations in genes such as GPAM and CIDEB is particularly intriguing [37,38,42,45]. These observations suggest that specific metabolic pathways may participate simultaneously in hepatocellular resilience and tissue adaptation during advanced disease.

The incorporation of clonal biology also conceptually aligns hepatology with other disciplines in which somatic evolution constitutes a central paradigm, including hematology and oncology. Age-related clonal hematopoiesis, for example, is now recognized as an independent determinant of cardiovascular risk and mortality [43,56].

Yet the clinical significance of the somatic component in MASLD remains uncertain. Its true prevalence, longitudinal stability, clonal threshold required to generate biological impact, and predictive value for fibrosis progression or hepatocarcinogenesis remain incompletely defined. Likewise, it remains unclear whether these alterations can be reliably detected through circulating cell-free DNA (cfDNA) in pre-neoplastic stages. Three interpretations remain open and cannot yet be adjudicated by available evidence: these somatic metabolic alterations (for example CIDEB and GPAM loss-of-function, or FOXO1 S22W) may represent an adaptive or protective hepatocellular response, a neutral marker of cumulative metabolic and regenerative stress, or an early contributor to clonal selection and the transition to hepatocellular carcinoma, the TERT promoter signal being the clearest pro-neoplastic example. With respect to cfDNA, detection in pre-cirrhotic disease is further constrained by the very low variant allele fractions expected from focal hepatic clones, the resulting limits of detection and specificity, the difficulty of distinguishing hepatic somatic clones from clonal haematopoiesis of indeterminate potential and other tissue-of-origin signals, and the absence of an established prognostic clonal burden threshold.

The incorporation of protective variants, polygenic models, and clonal biology has progressively transformed MASLD genetics from a framework focused exclusively on susceptibility into a substantially more complex one, with potentially major implications for the development of pharmacogenomic strategies.

## 3. From Genetic Risk to Biological Stratification: Proposal of the LS/CM/C Model (Table 3)

The convergence of germline genetics, polygenic risk scores, and clonal biology suggests that MASLD is not a uniform metabolic disorder but a constellation of partially differentiated biological trajectories. On this basis, we propose an interpretative framework composed of two predominant patterns—liver-specific and cardiometabolic—together with a potential acquired evolutionary component associated with advanced disease. Importantly, these patterns represent the two poles of a continuous polygenic gradient rather than a strict dichotomy. This is supported quantitatively by the two source studies: unsupervised clustering resolved the data into six native clusters, of which the liver-specific and cardiometabolic subtypes are only two (bootstrap Jaccard stability 0.73 [SD 0.07]; Calinski–Harabasz indices of 263, 174 and 18,774 in the discovery, validation and UK Biobank cohorts), with the two at-risk subtypes being histologically indistinguishable at baseline and carrying comparable chronic-liver-disease risk (adjusted HR 4.52 vs. 4.04) while diverging mainly in extrahepatic trajectory (cardiovascular HR 1.80 vs. 1.18; type 2 diabetes HR 6.82 vs. 2.91). In the partitioned polygenic-risk study, the dominant variant PNPLA3 loaded onto both clusters (90% of 1000 iterations resolved into two clusters), indicating a shared metabolic component. Mixed or intermediate profiles are therefore likely to be common, and the LS/CM bipartition is best read as a clinically focused synthesis of a broader six-cluster solution rather than a biological boundary.

**Table 3:** **The LS/CM/C framework on a single page.** A conceptual roadmap of the proposed model. The horizontal LS-CM axis is a germline continuum; the C axis is an orthogonal acquired clonal dimension that may superimpose on any point of it (operational proposal, not a third subtype).

	LS—Liver-Specific	CM—Cardiometabolic	C—Acquired Clonal (Proposed)
**Biology**	Hepatocyte-intrinsic lipid handling and remodelling	Systemic insulin resistance and adipose–liver axis	Somatic mutation under positive selection in chronic injury
**Genetics**	PNPLA3 I148M, TM6SF2 E167K, MBOAT7	GCKR P446L, FTO, INSR/COBLL1	FOXO1 S22W; somatic CIDEB and GPAM LoF; PKD1, KMT2D, ARID1A
**Phenotype**	Steatosis out of proportion to metabolic burden; lower CV risk	Central obesity, dyslipidemia, type 2 diabetes; higher CV risk	Clonal expansion in advanced fibrosis/cirrhosis; HCC transition
**Therapies**	THR-β—resmetirom (FDA accelerated approval 2024). PNPLA3 silencing—ASO/siRNA (investigational). HSD17B13 silencing—siRNA (investigational).	GLP-1R—semaglutide (FDA accelerated approval for MASH 2025). GIPR/GLP-1R—tirzepatide (approved for T2D/obesity; investigational in MASH). GIPR/GLP-1R/GCGR—retatrutide (investigational). Metabolic surgery (established).	No approved target. Conceptual: anti-FOXO1/anti-somatic-clone strategies (theoretical).
**Biomarkers**	Partitioned PRS (LS component); liver fat, MRI-PDFF	Partitioned PRS (CM component); metabolic profile	cfDNA somatic mutations as candidate predictive HCC biomarker

Geometry: a horizontal LS ↔ CM germline continuum, crossed by a vertical binary C axis that can overlay any position along it.

The first pattern corresponds to a predominantly hepatic phenotype, which we designate as LS (liver-specific). It is primarily driven by alterations involving hepatocellular lipid metabolism, lipid droplet remodeling, and VLDL secretion, particularly those related to PNPLA3, TM6SF2, and MBOAT7 [3,26,29]. Patients with a predominant LS profile may develop inflammation, fibrosis, or hepatocellular carcinoma that appears disproportionately severe relative to their systemic metabolic burden. In this context, intrinsic hepatocellular vulnerability constitutes the central biological axis of disease.

The second pattern corresponds to a predominantly cardiometabolic profile, designated CM (cardiometabolic). In this subgroup, visceral obesity, insulin resistance, type 2 diabetes, systemic inflammation, and increased cardiovascular risk predominate, together with variants involving GCKR, FTO, INSR, and COBLL1. From this perspective, the liver acts primarily as a target organ within a broader systemic metabolic disturbance.

This LS/CM framework allows reinterpretation of several clinical observations that have historically proven difficult to reconcile within purely metabolic models. Some patients develop advanced fibrosis or hepatocellular carcinoma despite only mild obesity—or even in the absence of overt metabolic syndrome—whereas others remain comparatively stable despite severe obesity. This dissociation suggests that systemic adiposity and hepatocellular vulnerability do not invariably evolve in parallel.

Nevertheless, the LS/CM dichotomy alone is likely insufficient to explain the biological complexity of advanced disease. Studies of hepatocellular clonality introduce a third dimension related to acquired evolutionary adaptation. On this basis, we propose incorporating a potential C (clonal) component, characterized by the expansion of hepatocytes harboring adaptive somatic mutations under chronic metabolic stress [38,46].

The C component does not represent a rigid subtype equivalent to LS or CM. Whereas LS and CM reflect predominant germline backgrounds, the clonal component constitutes a superimposed evolutionary dimension that can emerge upon either background during advanced fibrotic progression.

From a pharmacogenomic perspective, this model generates biologically plausible therapeutic hypotheses. Patients with predominant LS profiles may preferentially respond to therapies targeting hepatocellular metabolism or gene silencing—including resmetirom or strategies directed against PNPLA3 and HSD17B13—whereas patients with predominant CM profiles may derive greater benefit from systemic therapies targeting the broader metabolic axis, such as dual or triple incretin agonists [57,58].

In the context of the C component, the principal potential implications may relate to surveillance and prognostic stratification. If acquired clonal alterations could be reliably detected through circulating cell-free DNA (cfDNA) and shown to independently associate with fibrotic progression or hepatocellular carcinoma, they could eventually emerge as dynamic biomarkers of advanced disease. Clear precedents for such an approach already exist in oncology and clonal hematology [59,60].

The LS/CM/C model nevertheless remains a biological framework undergoing validation. At present, no pivotal clinical trial has prospectively demonstrated the clinical utility of partitioned PRS, LS/CM classification, or clonal biomarkers in MASLD. Likewise, it remains uncertain whether these patterns represent stable biological trajectories or dynamic states susceptible to modification, as previously discussed for PRS.

Accordingly, the aim of the LS/CM/C framework is not to establish an immediate new diagnostic taxonomy, but to offer an integrative structure that connects inherited genetics, metabolic heterogeneity, acquired somatic adaptation, and potential therapeutic response within a single model.

## 4. Pharmacogenomic Implications and Genotype-Directed Therapies (Table 4)

The emergence of biologically defined subtypes and targets validated through human genetics has coincided with the development of the first generation of genotype-directed therapies in MASLD. This transition is particularly significant because, for the first time in metabolic hepatology, part of the therapeutic pipeline no longer derives exclusively from animal models or biochemical hypotheses, but directly from observations grounded in human genetics. In this context, genetics ceases to be merely descriptive and begins to function as a tool for pharmacological prioritization.

The two principal platforms currently employed are antisense oligonucleotides (ASO) and small interfering RNA (siRNA) therapeutics [61,62]. Both strategies aim to reduce expression of specific genes within the hepatocyte, although they differ in mechanism of action, pharmacokinetics, and molecular flexibility.

ASO are synthetic single-stranded sequences that bind messenger RNA through base complementarity and promote degradation via RNase H1. They may act both within the nucleus and the cytoplasm, allowing modulation of splicing and post-transcriptional regulation. By contrast, siRNA are short double-stranded molecules that induce gene silencing through the RISC-Ago2 complex within the cytoplasm. Conjugation with N-acetylgalactosamine (GalNAc) facilitates selective hepatocyte uptake via the ASGPR receptor, thereby increasing hepatic specificity and prolonging therapeutic activity [63].

Both platforms have demonstrated profound reductions in hepatic messenger RNA expression in early-phase studies. AZD2693 achieved an approximately 89% reduction in hepatic PNPLA3 mRNA in paired liver biopsies [11], whereas ARO-HSD achieved reductions of 93.4% for HSD17B13 [13]. Overall, siRNA platforms tend to provide greater durability of effect owing to intracellular RISC stability, whereas ASO retain greater flexibility for molecular design and transcriptomic modulation [64].

**Table 4:** **Pharmacogenomic and genotype-directed therapeutic landscape in MASLD.** Status as of May 2026. Genotype-directed and genomically informed therapeutic landscape organised by development stage and by alignment with the LS/CM/C framework. ↑: increase; ↓ decrease.

**A. Clinically Active Genotype-Directed Therapies**																
**Target/Agent**			**Modality**		**Phase**			**NCT/Publication**		**Key Result**			**2026 Status**			**References**
PNPLA3 (I148M risk)—AZD2693/ION839			ASO GalNAc		Phase 1 (publ.)/2b FORTUNA discontinued			NCT05809934		−89% PNPLA3 mRNA; ↓ MRI-PDFF dose-dependent; insufficient clinical efficacy			DISCONTINUED Q3 2025 (AstraZeneca)			[11]
PNPLA3 (I148M)—JNJ-75220795/ARO-PNPLA3			siRNA GalNAc		Phase 1 (publ.)/transition to phase 2			—		↓ hepatic fat content in I148M homozygotes			LICENSED TO MADRIGAL 2026			[65,66]
HSD17B13—GSK4532990/ARO-HSD			siRNA GalNAc		Phase 1/2a (publ.)/2b HORIZON active			NCT05583344 (HORIZON); NCT06104319 (SKYLINE alcoholic)		−93.4% HSD17B13 mRNA; −42.3% ALT day 71			ACTIVE PHASE 2b			[13]
HSD17B13—rapirosiran/ALN-HSD			siRNA GalNAc		Phase 1 (publ. J Hepatol 2025)			NCT05519475 (NASHGEN-2 phase 2)		Robust HSD17B13 mRNA reduction with paired biopsy; acceptable safety			ACTIVE PHASE 2			[12]
PNPLA3 (I148M)—ALN-PNP/pivicasiran			siRNA GalNAc		Phase 1 SAD; phase 1/1b; phase 2a			NCT05648214; NCT06024408; NCT07527910		Phase 2a in homozygotes with/without GLP-1/GIP			ACTIVE			[67]
PNPLA3 (I148M)—LY3849891			siRNA GalNAc		Phase 1			NCT05395481		Safety, PK/PD, hepatic MRI			ACTIVE			[67]
PNPLA3 148M—AMG 609			siRNA		Phase 1 (completed)			NCT04857606		Phase 1 completed			LIMITED EVIDENCE			[67]
PNPLA3—PF-07853578			Oral small molecule		Phase 1 (volunteers)			NCT05890105		Proof of orally bioavailable anti-PNPLA3 small molecule			ACTIVE			[67]
TM6SF2, MBOAT7, others—multiple early-stage programmes			Various		Preclinical/phase 1			—		Indirect biopharmaceutical exploration of the LS axis			EXPLORATORY			[7,67]
PNPLA3 + GLP-1, others—combination programmes			Combined		Phase 2a			NCT07527910		Combination tested in trials			INITIAL EXPLORATION			[67]
**B. Emerging preclinical targets**																
**Target**			**Modality**		**Phase**			**NCT/publication**		**Key result**			**2026 status**			**References**
MTARC1 (LoF protective)			Various		Preclinical (no MASLD clinical programme)			—		↓ hepatic fat in human carriers			PRECLINICAL			[37,41]
GPAM (LoF protective)			Various		Preclinical			—		Protection against MASLD/cirrhosis in carriers			PRECLINICAL			[37]
CIDEB (LoF protective)			Various		Preclinical			—		53–54% protection against NAFLD/cirrhosis in carriers			PRECLINICAL—DUAL TARGET LS/C			[42,45]
**C. Stratification biomarkers and LS/CM/C implementation**																
**Programme**	**Target**			**Modality**			**Phase**		**NCT/publication**		**Key result**			**2026 status**	**References**	
Partitioned PRS	Two LS/CM scores			Stratification biomarker			Operational		Nat Med 2024		LS/CM bipartition with consistent profiles			AVAILABLE	[14]	
Clinical clustering	Two LS/CM clusters			Stratification algorithm			Operational		Nat Med 2024		Equivalent bipartition with 6 clinical variables			AVAILABLE	[15]	
cfDNA C component	Plasma somatic mutations			Predictive biomarker			Proposal/validation pending		—		Operational hypothesis			VALIDATION PENDING	[38,45,46,68]	
**D. Therapeutic alignment according to LS/CM/C biology**																
**Target subtype**		**Drug/programme**				**Mechanism**			**Regulatory status**			**Pharmacogenomic hypothesis**				**Reference**
LS		Resmetirom				Hepatocyte-specific TRβ selective agonist			FDA-approved March 2024 (MAESTRO-NASH)			Greater efficacy plausibly in LS-dominants (re-analysis pending)				[8,9,69]
LS		Rapirosiran/ALN-HSD				siRNA GalNAc anti-HSD17B13			Phase 2 NASHGEN-2; phase 1 published 2025			Mandatory selection in LS with genetic risk; HSD17B13 LoF best supported in 2025–2026				[12]
LS		GSK4532990/ARO-HSD				siRNA GalNAc anti-HSD17B13			Phase 2b HORIZON in F3 NASH; phase 2a SKYLINE in alcoholic disease			Selection in LS with genetic risk				[13]
LS		JNJ-75220795/ARO-PNPLA3				siRNA GalNAc anti-PNPLA3			Licensed to Madrigal 2026.			Selection by PNPLA3 I148M				[65]
LS		ALN-PNP/pivicasiran, LY3849891, AMG 609, PF-07853578				siRNA or small molecule anti-PNPLA3			Phase 1–2a (see Table 3)			Selection by PNPLA3 I148M				[67]
LS (discontinued)		AZD2693/ION839				ASO GalNAc anti-PNPLA3			Phase 1 published 2025; phase 2b discontinued Q3 2025			Proof of mechanism (89% mRNA reduction); insufficient clinical efficacy				[11]
CM		Semaglutide 2.4 mg				GLP-1 receptor agonist			FDA-approved August 2025 (ESSENCE)			Greater efficacy plausibly in CM-dominants (re-analysis pending)				[70,71]
CM		Tirzepatide				Dual GIP/GLP-1 agonist			Phase 2 SYNERGY-NASH positive; phase 3 SYNERGY-OUTCOMES (NCT07165028) ongoing			Greater efficacy plausibly in CM-dominants				[57]
CM		Retatrutide				Triple GIPR/GLP-1R/GCGR agonist			Phase 2 in MASH; phase 3 in progress			Plausible CM target				[58]
CM (cardiometabolic)		Metabolic surgery (RYGB, sleeve)				Anatomical-physiological intervention			Established for severe obesity			Mechanistically aligned with CM				[72]
LS preclinical		MTARC1, GPAM, CIDEB inhibitors				Hepatocyte-specific targeted therapies			Preclinical (no clinical programme in MASLD)			Future biopharmaceutical opportunity; CIDEB integrates LS + C				[37,41,42]
C (therapeutic gap)		Anti-FOXO1, anti-CIDEB somatic, cfDNA-C immunotherapy				Pharmacological category to be created			Theoretical; no programme			Operational hypothesis; prospective validation pending				[38,73]

### 4.1. PNPLA3: Therapies Targeting a Hepatocellular Pattern

PNPLA3 currently represents the principal genetic target in MASLD. Most anti-PNPLA3 programs selectively enroll carriers of the I148M variant—particularly homozygous individuals—thereby transforming genotype into an explicit inclusion criterion for clinical trials [11,66].

AZD2693/ION839 (AstraZeneca-Ionis), a GalNAc-conjugated ASO, achieved profound reductions in hepatic PNPLA3 mRNA together with dose-dependent reductions in liver fat quantified by MRI-PDFF [11]. However, the phase 2b FORTUNA trial was discontinued in 2025 because of insufficient efficacy [11]. This outcome introduced an important cautionary signal for the field: robust target engagement does not necessarily translate into clinically meaningful histological benefit. These levels of evidence should be kept distinct: the approximately 89% reduction in hepatic PNPLA3 mRNA derives from the phase 1 study, whereas FORTUNA was a subsequent phase 2b trial in F2–F3 disease. The dissociation between profound target engagement and clinical efficacy does not necessarily invalidate the target, but it cautions against assuming that profound molecular target engagement will translate into histological benefit.

The biological interpretation of the AZD2693 failure remains unresolved. Advanced fibrosis may be less reversible than initially anticipated; therapy may have been initiated too late in the disease course; or the duration of treatment may have been insufficient to detect histological improvement. It is also possible that modulation of a single hepatocellular pathway becomes inadequate once disease progression incorporates systemic inflammation, fibrotic remodeling, and acquired clonal adaptation. Recent studies on fibrotic plasticity and hepatocellular senescence support this possibility [74,75].

The anti-PNPLA3 pipeline also includes JNJ-75220795/ARO-PNPLA3, subsequently licensed to Madrigal, ALN-PNP/pivicasiran, LY3849891, AMG 609, and the oral small molecule PF-07853578 [76,77]. The emergence of oral compounds is particularly relevant because it demonstrates that functional modulation of PNPLA3 may extend beyond oligonucleotide-based platforms, with potentially important implications for cost, accessibility, and therapeutic scalability. In parallel, lipid-droplet-directed small molecules are emerging as a complementary therapeutic strategy in MASLD [78].

The biological rationale underlying these strategies aligns especially well with the LS pattern. The predominance of intrinsic hepatocellular mechanisms in LS patients may confer greater relative sensitivity to therapies centered on hepatocellular metabolism, including selective THR-β activation with resmetirom and PNPLA3 silencing strategies [9,14]. Although this hypothesis has not yet been prospectively validated, it provides a biologically plausible pharmacogenomic framework for interpreting future clinical trials.

### 4.2. HSD17B13: Pharmacological Reproduction of a Protective Phenotype

Unlike PNPLA3, therapeutic strategies directed against HSD17B13 aim to pharmacologically reproduce a naturally occurring protective loss-of-function variant first described by Abul-Husn et al. [39]. This approach probably constitutes one of the clearest examples of direct translation from human genetics into therapeutic development within hepatology.

At present, two GalNAc-conjugated siRNA programs have advanced into phase 2 clinical development. Rapirosiran/ALN-HSD demonstrated robust reductions in hepatic HSD17B13 mRNA together with an acceptable safety profile in paired biopsies at twelve weeks [12]. Similarly, GSK4532990/ARO-HSD achieved reductions of 93.4% in hepatic HSD17B13 mRNA together with significant decreases in ALT during phase 1/2a evaluation [13].

HSD17B13 currently represents the genotype-directed target with the most consistent clinical support in MASLD. In contrast to PNPLA3, where reduction in gene expression has not yet translated into unequivocal histological benefit, the development pathway for HSD17B13 has so far remained comparatively stable and coherent. The fact that these strategies are grounded in naturally occurring protective human variants provides an especially compelling biological rationale for continued pharmacological development.

### 4.3. Emerging Targets and Germline–Somatic Convergence

Beyond PNPLA3 and HSD17B13, several additional targets remain in advanced preclinical development. MTARC1 has been associated with lower hepatic fat accumulation, reduced ALT concentrations, and decreased liver-related mortality [37], whereas GPAM and CIDEB are of particular interest because they appear in both germline genetics and somatic clonal evolution [38,42,45].

CIDEB illustrates this convergence especially clearly. Germline loss-of-function variants significantly reduce the risk of fatty liver disease [42], whereas acquired somatic mutations affecting the same gene appear to confer adaptive advantages under conditions of chronic liver injury [45]. This overlap between germline protection and acquired clonal selection suggests that certain targets may simultaneously participate in susceptibility, disease progression, and tissue adaptation.

The situation partially resembles processes observed in oncology, where specific molecular alterations may exert different biological roles depending on the temporal and evolutionary context of the tissue [79]. In MASLD, the interaction between germline genetics and somatic evolution could eventually emerge as one of the principal determinants of advanced disease progression and therapeutic heterogeneity.

### 4.4. Genotype-Guided Trials and Pharmacogenomic Biomarkers

The incorporation of genotype as an inclusion criterion represents one of the most important recent methodological transitions in hepatology. Trials evaluating AZD2693, JNJ-75220795, and other anti-PNPLA3 strategies selected patients according to rs738409 genotype [11,76]. This approach reduces biological heterogeneity and increases statistical power, although it simultaneously limits generalizability.

The population-level implications are substantial because the frequency of PNPLA3 I148M differs markedly across ancestries [3]. Trials restricted to homozygous carriers therefore select biologically distinct populations that may become less representative unless explicit diversity strategies are incorporated. This issue has already been extensively discussed in genomic medicine and precision pharmacogenomics [40,80].

In parallel, the biomarkers employed in these programs are also evolving. The FDA and EMA currently accept paired histological endpoints—MASH resolution without worsening of fibrosis, and fibrosis improvement without worsening of MASH—as regulatory foundations for therapeutic approval [81,82]. However, genotype-directed therapies have additionally incorporated direct mechanistic biomarkers, including reductions in hepatic messenger RNA and MRI-PDFF measurements [11,12,13].

The next major step will likely involve integrating genetic and dynamic biomarkers within stratified clinical trials. These may include partitioned PRS, LS/CM classification, and—more speculatively—the detection of clonal alterations through circulating cell-free DNA (cfDNA). None of these biomarkers has yet been validated in a pivotal MASLD trial, but accumulated experience in oncology and cardiology demonstrates that such a transition is technically feasible [20,23].

## 5. Clinical Validation, Dynamic Biomarkers, and Future Priorities

The principal challenge facing the LS/CM/C framework is no longer conceptual, but clinical. The central question is not whether biological heterogeneity exists in MASLD—an observation already strongly supported by genetics, epidemiology, and metabolic phenotyping—but rather whether such heterogeneity reproducibly influences disease progression, therapeutic response, or hepatocellular carcinoma risk. Until these associations are prospectively validated, the model should be interpreted as a framework for biological organization rather than as an immediately applicable clinical classification.

The most immediate pathway toward validation will likely involve stratified reanalysis of completed clinical trials. Studies such as MAESTRO-NASH, ESSENCE, and SYNERGY-NASH contain sufficiently rich biological samples, metabolic phenotyping, and histological data to retrospectively apply partitioned PRS or LS/CM algorithms [9,57,70]. Such approaches could determine whether specific biological subgroups derive differential benefit from predominantly hepatocellular therapies as opposed to systemic metabolic interventions.

The same rationale could be extended to genotype-directed therapies targeting PNPLA3 and HSD17B13 [11,12,13]. To date, most of these programs have used genotype merely as an inclusion criterion; the next step will likely involve integrating more complex layers of information, including partitioned PRS, LS/CM classification, dynamic metabolic phenotypes, and biomarkers of fibrotic or clonal progression. This strategy could reduce part of the biological heterogeneity observed in current trials and progressively align MASLD with precision medicine models already established in oncology and molecular hematology [20,21].

The acquired clonal C component introduces challenges beyond those already discussed. It remains uncertain whether these alterations represent protective adaptive mechanisms, markers of accumulated tissue stress, or early events in neoplastic transformation.

The possibility of detecting somatic mutations through circulating cell-free DNA (cfDNA) constitutes one of the most compelling hypotheses in the field. In oncology, cfDNA analysis already permits detection of subclinical clones, monitoring of minimal residual disease, and dynamic assessment of tumor evolution [60,83]. In hepatology, a similar strategy could potentially allow monitoring of hepatocellular clonal expansion without the need for repeated biopsies. Nevertheless, no prospective studies currently validate cfDNA as a dynamic biomarker of progression in non-neoplastic MASLD.

This issue becomes particularly relevant in hepatocellular carcinoma surveillance. The sensitivity of ultrasound for early HCC detection in MASLD remains limited, especially in the setting of obesity and marked steatosis [84,85]. Furthermore, a substantial proportion of MASLD-associated hepatocellular carcinomas arise in the absence of advanced cirrhosis, complicating traditional surveillance strategies based exclusively on fibrosis staging [86,87].

In this context, integrating PRS, elastography, inflammatory biomarkers, and clonal signals derived from cfDNA could substantially improve risk stratification, although this possibility remains clearly exploratory.

Another major challenge concerns the population transferability of genetic models. Variants such as PNPLA3 I148M display profound differences in frequency across ancestries [3], and PRS developed predominantly in European populations show reduced performance in other populations [40,54]. This limitation is not unique to MASLD and already represents a recognized constraint of contemporary genomic medicine. Without robust multi-ancestry validation, there is a substantial risk of developing pharmacogenomic tools with unequal applicability across populations. From an implementation standpoint, routine genotyping or full PRS computation would add per-patient cost and require laboratory and bioinformatics infrastructure that is difficult to justify in a disease of very high prevalence and substantial socioeconomic burden, and established reimbursement pathways are currently lacking; deploying ancestry-biased PRS prematurely could widen rather than narrow these inequities. In this respect, the LS/CM/C framework should be positioned as a research-stage complement to, rather than a replacement for, the fibrosis-based stratification recommended by current AASLD and EASL–EASD–EASO guidance.

The regulatory dimension constitutes another area that remains insufficiently developed. At present, neither the FDA nor the EMA accepts PRS or genetic biomarkers as regulatory criteria in pivotal MASLD trials [19,82]. Nevertheless, precedents from oncology demonstrate that complex molecular platforms may progressively become incorporated into both clinical trial design and regulatory approval pathways [22,23]. MASLD could undergo a similar transition during the coming decade, particularly if genotype-directed therapies demonstrate reproducible clinical benefit within specific biological subgroups.

Finally, the development of large longitudinal cohorts will be essential for validating any biologically stratified framework. Population-based cohorts integrating genetics, metabolomics, liver imaging, histology, electronic health records, and long-term follow-up will allow evaluation of whether the LS/CM/C model provides genuine incremental value beyond conventional clinical tools. In this context, deeply phenotyped population platforms may prove especially valuable for linking genetics, clinical progression, and therapeutic response under real-world conditions.

Overall, the LS/CM/C model should currently be interpreted as a translational research framework rather than a definitive clinical classification. Its future utility will depend on three fundamental questions: whether it improves prediction of disease progression; whether it identifies reproducible differences in therapeutic response; and whether it provides clinically useful biomarkers for surveillance and risk stratification.

Until these questions are resolved, the model should be regarded as a structured biological hypothesis that still requires validation rather than an immediate tool for routine clinical practice.

The LS/CM axis may also extend to metabolic and alcohol-associated steatotic liver disease (MetALD). Alcohol-interacting loci such as ADH1B (already included in Table 1) provide a plausible genetic entry point for incorporating alcohol exposure into the framework, although genetic data in MetALD remain limited and preliminary, and any extension of the model to this population requires dedicated validation (Box 2).

Box 2 **Research agenda.**

(1) stratified re-analysis of MAESTRO-NASH and ESSENCE applying partitioned PRS and LS–CM algorithms to test for differential treatment response;
(2) prospective evaluation of cfDNA somatic-clone burden as a predictor of fibrosis progression and hepatocellular carcinoma in deeply phenotyped longitudinal cohorts;
(3) multi-ancestry re-derivation and validation of the partitioned PRS.

## 6. Conclusions

The genetics of MASLD has evolved from a susceptibility-centered paradigm toward a progressively more biologically stratified and pharmacogenomic vision of disease. The integration of germline variants, protective alleles, polygenic models, and somatic clonal adaptation indicates that MASLD is not a single, biologically uniform metabolic disorder but a set of partially distinct disease trajectories.

On this basis, we propose an interpretative framework composed of two predominant patterns—liver-specific (LS) and cardiometabolic (CM)—together with a potential acquired evolutionary component associated with advanced disease progression (C). The LS pattern reflects intrinsic hepatocellular vulnerability driven predominantly by alterations in lipid metabolism, whereas the CM pattern is more coherently integrated within the systemic cardio-kidney-metabolic continuum [14,15,16,17].

The emergence of therapies directed against PNPLA3 and HSD17B13, together with the accelerated approval of resmetirom and semaglutide, further reinforces the biological plausibility of this transition and progressively aligns MASLD with precision medicine models already established in oncology and molecular hematology [8,9,11,12,71].

Nevertheless, the LS/CM/C framework still requires validation. Important limitations persist, particularly regarding prospective testing, the ancestral transferability of PRS, and the absence of standardized dynamic biomarkers [40,54].

The convergence of human genetics, targeted pharmacology, and somatic evolution suggests that the field is shifting toward a hepatology defined less by the amount of hepatic fat and more by the specific biological mechanisms that drive disease in each individual patient.

## Data Availability

No new data were created or analyzed in this study. Data sharing is not applicable to this review.

## Abbreviations

AASLD, American Association for the Study of Liver Diseases; ABHD5/CGI-58, abhydrolase domain-containing 5/comparative gene identification-58; ADH1B, alcohol dehydrogenase 1B; ALT, alanine aminotransferase; ASGPR, asialoglycoprotein receptor; ASO, antisense oligonucleotide; ATGL, adipose triglyceride lipase; bNMF, Bayesian non-negative matrix factorization; C, clonal; cfDNA, circulating cell-free DNA; CIDEB, cell death-inducing DFFA-like effector B; CKM, cardio-kidney-metabolic; CM, cardiometabolic; COBLL1, cordon-bleu WH2 repeat protein-like 1; CVD, cardiovascular disease; EASD, European Association for the Study of Diabetes; EASL, European Association for the Study of the Liver; EASO, European Association for the Study of Obesity; EMA, European Medicines Agency; FDA, Food and Drug Administration; FOXO1, forkhead box O1; FTO, fat mass and obesity-associated gene; GalNAc, N-acetylgalactosamine; GCGR, glucagon receptor; GCKR, glucokinase regulator; GIP, glucose-dependent insulinotropic polypeptide; GIPR, glucose-dependent insulinotropic polypeptide receptor; GLP-1, glucagon-like peptide-1; GLP-1R, glucagon-like peptide-1 receptor; GPAM, glycerol-3-phosphate acyltransferase (mitochondrial); GWAS, genome-wide association study; HCC, hepatocellular carcinoma; HR, hazard ratio; HSD17B13, hydroxysteroid 17-beta dehydrogenase 13; INSR, insulin receptor; LDL, low-density lipoprotein; LoF, loss-of-function; LS, liver-specific; MASH, metabolic dysfunction-associated steatohepatitis; MASLD, metabolic dysfunction-associated steatotic liver disease; MBOAT7, membrane-bound O-acyltransferase domain-containing 7; MetALD, metabolic and alcohol-associated steatotic liver disease; MRI-PDFF, magnetic resonance imaging proton density fat fraction; MTARC1, mitochondrial amidoxime-reducing component 1; NAFLD, non-alcoholic fatty liver disease; NASH, non-alcoholic steatohepatitis; NCAN, neurocan; OR, odds ratio; PNPLA3, patatin-like phospholipase domain-containing 3; PRS, polygenic risk score; RISC, RNA-induced silencing complex; SD, standard deviation; siRNA, small interfering RNA; T2D, type 2 diabetes; TERT, telomerase reverse transcriptase; THR-β, thyroid hormone receptor beta; TM6SF2, transmembrane 6 superfamily member 2; VLDL, very-low-density lipoprotein; VLDL1, large triglyceride-rich very-low-density lipoprotein.
